# Refractive indices of layers and optical simulations of Cu(In,Ga)Se_2_ solar cells

**DOI:** 10.1080/14686996.2018.1458579

**Published:** 2018-05-15

**Authors:** Romain Carron, Enrico Avancini, Thomas Feurer, Benjamin Bissig, Paolo A. Losio, Renato Figi, Claudia Schreiner, Melanie Bürki, Emilie Bourgeois, Zdenek Remes, Milos Nesladek, Stephan Buecheler, Ayodhya N. Tiwari

**Affiliations:** a Laboratory for Thin films and Photovoltaics, Empa – Swiss Federal Laboratories for Materials Science and Technology, Dübendorf, Switzerland; b Institute of Computational Physics, Zurich University of Applied Sciences (ZHAW), Winterthur, Switzerland; c Laboratory for Advanced Analytical Technologies, Empa – Swiss Federal Laboratories for Materials Science and Technology, Dübendorf, Switzerland; d Institute for Materials Research (IMO), Hasselt University, Diepenbeek, Belgium; e IMOMEC Division, IMEC, Diepenbeek, Belgium; f Institute of Physics, Academy of Sciences of the Czech Republic, Prague, Czech Republic

**Keywords:** Cu(In,Ga)Se_2_, refractive index, optical simulations, thin films, solar cells, absorption losses, carrier collection losses, optical losses, 50 Energy Materials, 209 Solar cell / Photovoltaics, 306 Thin film / Coatings, 204 Optics / Optical applications

## Abstract

Cu(In,Ga)Se_2_ based solar cells have reached efficiencies close to 23%. Further knowledge-driven improvements require accurate determination of the material properties. Here, we present refractive indices for all layers in Cu(In,Ga)Se_2_ solar cells with high efficiency. The optical bandgap of Cu(In,Ga)Se_2_ does not depend on the Cu content in the explored composition range, while the absorption coefficient value is primarily determined by the Cu content. An expression for the absorption spectrum is proposed, with Ga and Cu compositions as parameters. This set of parameters allows accurate device simulations to understand remaining absorption and carrier collection losses and develop strategies to improve performances.

## Graphical abstract

**Figure:**
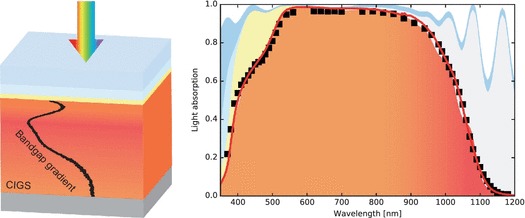

## 1. Introduction

Polycrystalline Cu(In,Ga)Se_2_ (CIGS) has gained a significant interest as a light absorber for high-efficiency photovoltaic devices. Record efficiencies for solar cells based on co-evaporated CIGS are 22.6% [1] for high-temperature process on glass and 20.4% [2] for low-temperature process on polyimide substrate. Complementary to empirical optimization approaches, optical simulations may guide efforts toward minimization of optical reflection and absorption losses, and toward optimization of the light absorption. Comparison of simulations with experimental data can also deliver insights into the carrier collection losses in devices. However, a reliable optical model is required in order to extract useful information from simulations.

A comprehensive characterization of the different layers in a CIGS solar cell was for example reported by Hara et al*.* [3]. Further data and discussions are also available for the different solar cell layers: magnesium fluoride antireflective coating (MgF_2_) [4], Al-doped zinc oxide (ZnO:Al) [5–7], highly resistive zinc oxide (ZnO) [8] and references therein, cadmium sulfide (CdS) [4], metallic molybdenum (Mo) [9], and molybdenum selenide (MoSe_x_) [10,11] which spontaneously forms at the Mo/CIGS interface during CIGS deposition [12].

The CIGS absorber bandgap can be adjusted by tuning the composition ratio GGI defined as [Ga]/([Ga] + [In]). CIGS layers for high-efficiency solar cells typically exhibit a double compositional Ga grading, with GGI highest at the back contact and lowest below the upper interface [13,14]. The CIGS chalcopyrite phase can accommodate some degree of Cu deficiency characterized with the CGI ratio defined as [Cu]/([Ga] + [In]), which is typically between 0.8 and 0.9 for high-efficiency absorbers [1,2,15]. On the other hand, excess Cu tends to segregate as CuSe_x_ alloys detrimental to the device properties. Detailed phase diagrams of CuInSe_2_, CuGaSe_2_ , and CuInGaSe_2_ materials are reported elsewhere [16–19].

In the last decades, the dielectric function of CIGS was reported in a number of publications [20–26]. Because of the absorber compositional grading, the dielectric function must be known for any composition in order to predict the external quantum efficiency (EQE). The Ga content is of general interest as it determines the bandgap, but the influence of Cu was often overlooked in previous studies [3,20,26]. For devices with absorber thicknesses above 1 μm the EQE is crucially determined by the absorption coefficients at photon energies just above the optical absorption edge. The preferred characterization technique is often ellipsometry. However, the data treatment relies on a fit of a wide energy range using a small number of oscillators, and especially for thin layers the fitting procedure might lack sensitivity to low absorption coefficients. As an example, Alonso et al*.* [22] has not reported absorption coefficients values lower than *k* = 0.1 for lack of confidence in experimental data (corresponding to *α* around 1.2 × 10^4^ cm^−1^). More recently, Minoura et al*.* [24] reported a Ga and Cu composition-dependent dielectric function for CIGS. However, further work is required to refine those results, especially owing to the low number of investigated samples, to their nature (around 50 nm thick on Si substrates), and to the uncertainty on the compositions of thin layers.

In this contribution, we present characterization results on layers deposited under conditions as close as possible to that of high-efficiency devices. The paper is organized as follows.

The optical refractive indices of the front and back contact layers of a standard CIGS solar cell are determined by combining ellipsometry, reflectance, and transmittance measurements. Model parameters to the dielectric functions are derived for Mo, MoSe_x_, CdS, non-intentionally doped ZnO, ZnO:Al, and MgF_2_ materials. The discrepancies with available datasets are discussed.

Then the optical absorption of the CIGS material is determined from reflectance and transmittance measurements on absorber layers transferred onto transparent substrates. The focus is placed on the energy range in the vicinity of the bandgap, essential to determine the shape of the EQE curve. After a careful composition calibration, the influence of the Ga and Cu contents on the optical absorption spectrum is characterized in terms of bandgap, absorption intensity, and sub-bandgap absorption tail. Alternative techniques provide additional inputs for the sub-bandgap absorption tail. An expression is proposed for the optical absorption of CIGS as function of the Cu and Ga contents. A comparison with literature data reveals significant differences close to the bandgap region, affecting the shape of simulated EQE spectra.

Finally, optical numerical simulations are performed using GGI depth profiles of CIGS layers as an input. A comparison of simulated reflectance and EQE curves with experimental data allows discriminating the carrier collection losses from incomplete absorption losses. An alternative procedure to do so is developed, where optical measurements on absorbers transferred onto transparent substrates are required instead of simulations based on depth profiles. Possible gains in the short circuit currents are discussed in terms of GGI grading.

## 2. Experimental details

Each of the layers composing a CIGS solar cell was deposited on a soda –lime glass (SLG) substrate. ZnO, ZnO:Al, and MgF_2_ were also deposited on (100)-oriented Si wafer substrates covered with native oxide. Various deposition techniques were used: RF magnetron sputtering (ZnO, ZnO:Al with target composition 2% Al_2_O_3_ by weight), DC magnetron sputtering (Mo), e-beam evaporation (MgF_2_), chemical bath deposition (CBD) (CdS), co-evaporation (CIGS). The CdS CBD process is described elsewhere [2]. Uniform CdS growth on SLG substrates was prompted with a thin (around 4 nm) seed layer deposited by sputtering from a CdS target. The duration of the CBD process was slightly reduced as compared to a standard CdS deposition in order to reduce as much as possible the adhesion on the surface of CdS nanocrystals produced by homogeneous nucleation in the CBD solution. Only the MoSe_x_ layers were obtained under very different conditions as the layers in a solar cell: thin Mo layers were selenized at 600 °C nominal in a rapid thermal process (RTP) system (Annealsys AS-ONE), using a 500 mbar N_2_ atmosphere in presence of Se.

The CIGS layers were deposited by single-stage co-evaporation at constant temperature on SLG substrates coated with a SiO_x_ alkali diffusion barrier and an around 500 nm thick Mo back contact. Additionally, a CuInSe_2_ and a CuGaSe_2_ layer were grown according to a three-stage procedure at constant temperature. The depositions were performed in two different reactors, both able to produce multistage graded absorbers with a typical efficiency above 19% after device completion including anti-reflection layer. The nominal substrate temperatures were between 400 and 520 °C.

The transfer of CIGS layers on transparent substrates consisted in a mechanical peeling off using a SLG substrate glued on the CIGS absorber. The procedure is described in the Supplemental information.

Reflectance and transmittance measurements were performed using a Shimadzu UV-vis, Kyoto, Japan 3600 spectrophotometer equipped with an integrating sphere, while correcting for instrumental responses stemming from diffuse and specular reflections on the sample and on the reflectance standard. Measurements were typically carried out in a wavelength range from 300 to 2000 nm. Hereafter, the absorptance is defined from reflectance (*R*) and transmittance (*T*) curves as Abs = 1 – *R* – *T*.

The surface roughness was characterized by atomic force microscopy (AFM) measurements using a NanoSurf-AFM, Liestal, Switzerland Mobile S instrument with experimental noise of 0.1 nm RMS in the vertical direction. The sample AFM roughness was evaluated as the *R*_*q*_ roughness averaged over several 2 × 2 μm^2^ and 5 × 5 μm^2^ topography images.

Layer thicknesses were determined using a KLA-Tencor Corporation, Milpitas, California, US profilometer. For the layers with thickness below 100 nm this was confirmed by cross-section scanning electron microscopy (SEM) imaging using a Hitachi S-4800, Chiyoda, Tokyo, Japan using 5 keV acceleration voltage. The calibration of the SEM magnification is regularly checked against a standard. The same instrument was used for EDX characterization (20 keV).

The composition of the CIGS layers was determined based on the intensities of the Cu, Ga, and In characteristic K lines in X-ray fluorescence (XRF) measurements. Inductively coupled plasma optical emission spectroscopy (ICP-OES) measurements were also conducted and experimental details are reported in the Supplemental information.

Compositional depth profiling was performed by time-of-flight secondary ion mass spectrometry (ToF-SIMS, ION-TOF, GmbH, Münster, Germany TOF-SIMS5 measurement unit). GGI depth profiles where computed from the Ga-71 and In-113 traces, which were scaled according to the integral GGI composition.

EQE, photocurrent spectroscopy (PCS), and photothermal deflection spectroscopy (PDS) measurements were performed according to standard procedures. Details are given in the Supplemental information, as well as in Refs. [27,28] for the PDS setup.

The optical data were processed using the RefFit software [29] which allows for simultaneous fitting of reflectance, transmittance, and ellipsometry data based on a unique multilayer model. When available, data acquired on layers deposited on both SLG and Si substrates were fitted simultaneously using the same multilayer model.

Optical models were constructed for multilayers as SLG/material/roughness layer, or Si/Si–SiO_2_ intermediate layer/SiO_2_/material/roughness layer depending on substrate. The refractive indices of Si, Si–SiO_2_ intermediate layer, and SiO_2_ were taken from literature [30]. Thicknesses of material layers determined by profilometry or SEM were used as inputs, and only allowed to vary by a few percent during the last fit refinements. The roughness layer was modeled as a Bruggeman effective medium approximation (EMA) layer [31] composed of the material and void in a 50–50% mixture. The roughness layer thickness was fixed to 5x the AFM RMS roughness *R*_*q*_, similarly as in Ref. [32]. The roughness layer thicknesses were below the generally accepted validity of this approximation (max 1/10 of the optical wavelength).

The materials dielectric functions were constructed as the sums of Lorentz and Tauc-Lorentz oscillators on top of a constant *ɛ*_∞_ ensuring consistency with Kramers–Kronig relations. Lorentz oscillators are described with the parameters eigenfrequency *ω*_0_, plasma frequency *ω*_*p*_ and linewidth *γ*, whereas Tauc-Lorentz oscillators require an additional amplitude parameter *Amp*.

## 3. Front and back contact dielectric functions

**Figure 1.**
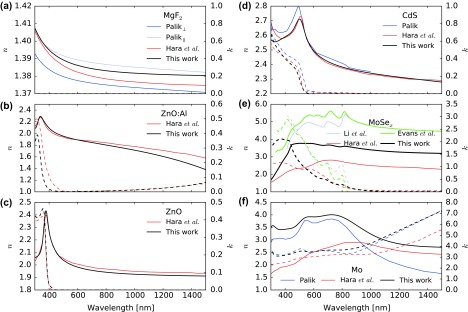
Wavelength-dependent refractive indices of layers determined in this work, together with data from Refs. [3,4,9–11]. Continuous lines indicate the real part *n* and refer to the left ordinate, and dashed lines indicate the imaginary part *k* and refer to the right ordinate. (a) Refractive index of MgF_2_ antireflective layer, (b) ZnO:Al layer, (c) ZnO highly resistive layer, (d) CdS buffer, (e) MoSe_x_ interlayer, and (f) Mo back contact.

Dielectric functions are often determined by ellipsometry measurements only. We observed that consideration of reflectance and transmittance data adds a significant constraint on the fits, especially regarding the absorption coefficient. For each material, the resulting dielectric functions are shown in Figure 1, while the raw reflectance, transmittance, and ellipsometry spectra and fits of samples with SLG substrates are displayed in Figure SI.2.

**Figure 2.**
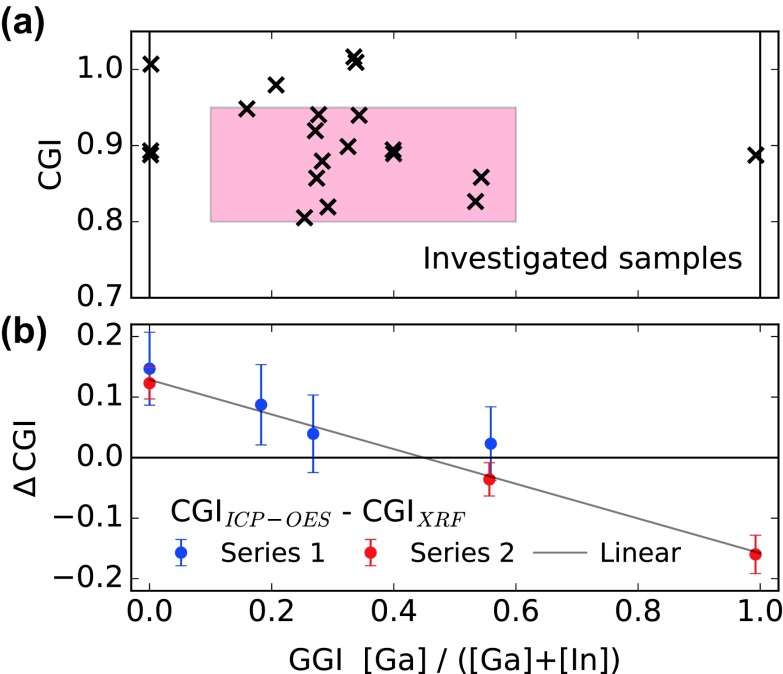
(a) Graphical summary of the different sample compositions investigated in this study. The colored area corresponds to the typical compositional range of high-quality CIGS absorber layers. (b) Difference in CGI composition as measured by ICP-OES and XRF, as function of the GGI.

Note: The continuous line represents the proposed linear correction.

A SLG plate similar to those used as substrates was characterized following the procedure described above, and the resulting dielectric function was used as input to the modeling of the other material layers.

**Figure 3.**
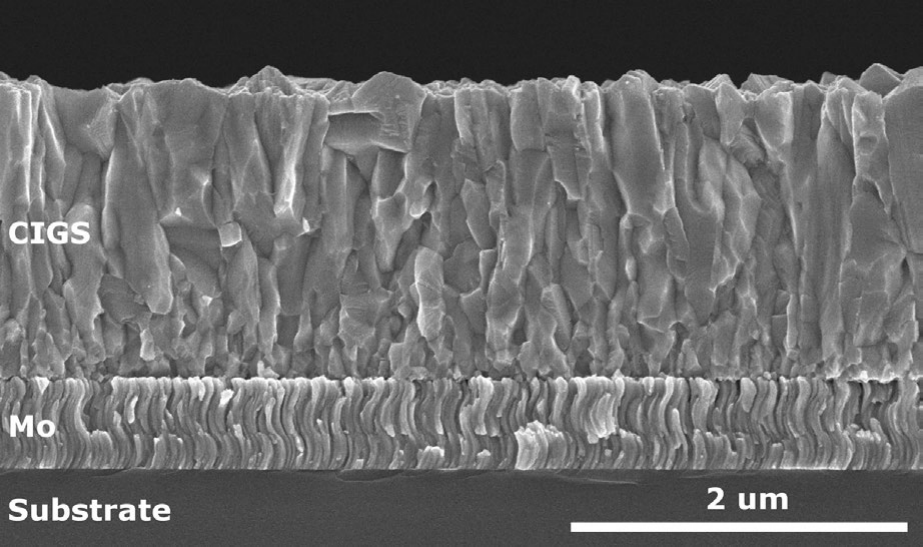
SEM cross-section micrograph of a typical investigated single stage CIGS (GGI 0.16, CGI 0.95).

Note: Here, the alkali diffusion barrier cannot be distinguished from the substrate.

The properties of the Mo layers with thicknesses between 13 and 18 nm deposited on SLG substrates, along with a thicker, opaque layer measured in reflection mode only. The Mo layers grown at Empa exhibit a columnar, bended structure visible in SEM micrographs (Figure 3), originating from successive deposition steps with moving the substrate in front of the target. Ellipsometry data appeared to depend on the sample orientation. Data acquisitions were thus performed by placing the samples such that the preferential direction of the crystallites was at 45° with respect to the incoming light beam. This orientation delivers ellipsometry values intermediate between parallel and perpendicular relative orientations. The intricate shape of the metallic Mo dielectric function was determined according the following procedure. An initial guess was obtained by fitting the data-set published by Palik [9] with a set of Lorentz oscillators. The experimental data were then fitted while keeping fixed the eigenfrequencies *ω*_0_. In a second step, an oscillator was added at high frequency, and the eigenfrequency *ω*_0_ of another oscillator was varied at low frequency. The material properties appeared to depend on the process conditions. Thus, an experimental error can be estimated from the spread in the best fits to layers deposited under slightly different conditions. This can be described as a shift in values roughly independent from wavelength over the visible to NIR range, and amounts to ${}_{-0.2}^{+0.1}$ on *n* and to ${}_{-0.5}^{+0.3}$ on *k*. Our values notably present broader features than those reported in Ref. [9], possibly due to the columnar nanostructure of the investigated layers.

The dielectric function of approximatively 110 nm thick MoSe_x_ layers was fitted using a single Tauc-Lorentz transition in the visible range, decorated with a set of Lorentz oscillators to reproduce the relatively sharp features observed in the ellipsometry, reflectance, and transmittance spectra. The fit somewhat differs from the experimental data (see Figure SI.2). In addition to the model simplicity, the surface roughness generated during the selenization process is also problematic for an accurate modeling of the data. We report markedly lower *n* and *k* values as compared to Refs. [10,11], and the spectral features are less sharp. The MoSe_x_ growth process was markedly different from that occurring in an actual device, thus the material properties might actually differ. Especially, morphology changes are possible at the nanoscale (layered vs. disordered), and contamination from the substrate cannot be excluded due to the high temperature involved during preparation. Especially Na may diffuse from the SLG substrate, which is known to promote the formation of MoSe_x_ layers [33,34].

CdS layers with approximatively 16 and 27 nm thicknesses were grown on seeded SLG substrates. Significantly, less absorption was observed below the bandgap as compared to literature data-sets [3,9].

A good fit to the optical data of highly resistive ZnO layers was achieved with a combination of two Tauc-Lorentz oscillators describing the bandgap. Reflectance and transmittance evidenced no optical absorption above 500 nm. A good agreement is obtained with comparable literature reports [3,8]. A fit to the low energy data with no Tauc-Lorentz oscillator leads to a value of *ɛ*_∞_ = 3.71, in line with reported values [8].

The dielectric function of Al-doped ZnO was described with a model similar to that of ZnO, with an additional Lorentz oscillator at low frequency accounting for the free carrier absorption. A good fit to the data was achieved. We report a better transparency close to 400 nm than Ref. [3], while maintaining a comparable level of absorption in the infrared. We stress that the layer properties, especially the infrared absorption and the optical bandgap, are significantly affected by target composition and process conditions. Accurate data-sets should be obtained from process-relevant layers and not from literature. When the free carrier concentration in a TCO increases, the optical bandgap is widened due to the combined effect of the Burstein –Moss shift [35,36] and the electron –electron repulsive interaction [37–39]: in our case a blueshift of 0.31 eV as compared to non-intentionally doped ZnO was determined from the Tauc plot method. A fit to the data with energy below 1.85 eV using a single Lorentz oscillator model assuming *ω*_0_ = 0 cm^−1^ results in a value of *ɛ*_∞_ = 3.75, in line with usual values [7]. Last, we determine the carrier density and intra-grain mobility by following the formalism of Refs. [5,40]. As detailed in the Supplemental information, we obtain a carrier density in reasonable agreement with Hall measurements, and an intra-grain mobility higher than Hall value, similarly as reported in Ref. [41].

The MgF_2_ birefringent material was assumed anisotropic as we could not evidence a dependency of the ellipsometry data on the sample orientation. No optical absorption at any wavelength could be evidenced in the layers investigated. No physical interpretation should be drawn from the MgF_2_ model parameters reported in Table 1, as more reasonable frequencies would fit the data almost as well as those best fit values.

**Table 1.** Summary of the parameters of the dielectric functions, for each material investigated in this study.

Material	Oscillator	*ω*_0_ [cm^−1^] or *ɛ*_∞_	*ω*_*p*_ [cm^−1^]	*γ* [cm^−1^]	*Amp* [cm^−1^]
SLG	*ɛ*_∞_	1.94			
	Lorentz	30879	17.068	2226.9	
	Tauc-Lorentz	54567	30067	88.575	86254
Mo	*ɛ*_∞_	2.38			
	Lorentz	42216	38460	4887.7	
	Lorentz	38687	43451	8517.4	
	Lorentz	33975	56483	7781	
	Lorentz	27247	92399	21946	
	Lorentz	19236	27629	6059.7	
	Lorentz	14398	43866	9235.8	
	Lorentz	6977.2	34394	9270.7	
	Lorentz	0	59958	1120.1	
MoSe	*ɛ*_∞_	3.48			
	Lorentz	22939	10000	1936	
	Lorentz	17426	5386	2824	
	Lorentz	14535	5411	2016	
	Lorentz	12452	3437.5	1210	
	Tauc-Lorentz	24138	8484.9	16271	462365
CdS	*ɛ*_∞_	3.11			
	Lorentz	0	3544.6	0	
	Tauc-Lorentz	19712	18243	3699.8	311993
	Tauc-Lorentz	26941	17021	36014	465139
	Tauc-Lorentz	37767	20947	8278.8	124550
ZnO	*ɛ*_∞_	1.56 (3.71 with no Tauc-Lorentz)			
	Tauc-Lorentz	26674	24782	2833.3	665906
	Tauc-Lorentz	45608	22064	91216	704301
ZnO:Al	*ɛ*_∞_	2.01 (3.75 with no Tauc-Lorentz)			
	Lorentz	2500	8036.6	607.22	
	Tauc-Lorentz	30591	23279	5087	120859
	Tauc-Lorentz	47771	18579	10593	190444
MgF_2_	*ɛ*_∞_	0			
	Lorentz	166990	230323	0	

Notes: *Amp* is the additional parameter specific to the Tauc-Lorentz oscillators. For comparison with literature data, an additional value of *ε*_∞_ is provided for ZnO and ZnO:Al, obtained by fitting the low-energy data with Lorentz oscillators only.

## 4. Optical absorption in CIGS with varied compositions

A set of around 2 μm thick CIGS layers was deposited with GGI and CGI compositions spanning the range of interest (typical metal ratios of high quality devices), as depicted in Figure 2(a). ToF-SIMS measurements did not evidence any non-uniformity in the GGI depth profile. Details about the samples are reported in Table 2.

**Table 2.** Summary of the characteristics of the samples reported in this study.

	GGI	CGI	Thick. [μm]	Depos. *T* [°C]	Stages	*E*_*g*_ [eV]	*A* [cm^−1^]	Exp. decay energy [meV]	Urbach energy [meV]	
									PCS	PDS
	0.00	0.89	2.00	520	1	1.004	53324	39.2		
	0.00	0.98	1.95	520	1	1.006	60941	24.2		
^B^	0.00	0.89	2.50	400	3	1.006	64825	36.3		
	0.16	0.95	1.99	413	1	1.108	69620	29.7		
	0.21	0.98	1.76	520	1	1.143	78935	23.9		
	0.25	0.81	1.89	413	1	1.173	51497	50.8		
	0.27	0.92	1.80	520	1	1.180	67869	41.1		26
	0.27	0.86	1.74	413	1	1.181	59290	44.1		
	0.28	0.94	1.89	413	1	1.177	72955	33.9		
	0.28	0.88	1.72	413	1	1.185	62381	46.0		31
	0.29	0.82	1.56	520	1	1.191	61569	49.6		
^etch^	0.29	0.82	1.25	520	1	1.198	58483	40.3		
^B^	0.33	0.90	2.28	400	1	1.208	83358	51.6	20	
^B^	0.33	1.02	2.33	400	1	1.218	103355	19.5	23	25
^B^	0.34	1.01	2.26	400	1	1.222	95843	40.9	22	
^B^	0.34	0.94	2.28	400	1	1.218	80942	40.5	20	26
	0.40	0.89	1.94	413	1	1.258	69729	39.7		
	0.40	0.89	1.92	520	1	1.264	74089	44.4		
^back^	0.40	0.89	1.92	520	1	1.253	69062	45.2		
	0.53	0.83	1.83	413	1	1.335	61506	53.7		
	0.54	0.86	1.85	413	1	1.349	68264	55.6		
	0.99	0.89	1.73	413	3	1.660	96919	18.9		
^back^	0.99	0.89	1.73	413	3	1.656	103657	20.8		

Notes: GGI and CGI compositions are given after correction deduced from ICP-OES measurements. Deposition temperatures are nominal: processes are compatible with polyimide substrates at 413 °C nominal in reactor A and 350 °C in reactor B. Symbol ^B^: deposition performed in reactor B. Symbol ^etch^: Br etching prior transfer onto transparent substrate. Symbols ^back^: reflectance, transmittance measurements from substrate side.

This section is organized as follows. First the determination of the sample composition is discussed, then the measurement artifacts are discussed. Finally after data processing, an expression is established for modeling the composition-dependent absorption coefficient of CIGS, which is valid for compositional ranges of the GGI between 0 and 1 and of the CGI between 0.75 and 1.

### 4.1. Layer composition characterization

Our group typically reports XRF compositions calibrated against a graded reference sample with composition similar to high-efficiency devices. However, a good accuracy is only achieved in a certain range close to the composition of the reference: as an example this calibration markedly underestimates the [Cu]/[In] composition ratio of CuInSe_2_ layers. ICP-OES is an alternative to XRF, in principle more accurate although destructive and unsuited for routine characterization.

A correction function is established to the non-contact XRF characterization by characterizing two series of ungraded absorbers using both ICP-OES and XRF. Compositions comparison revealed a systematic underestimation of XRF GGI values by 3% relative, i.e. an underestimation by 0.01 absolute for standard compositions. Comparison of the CGI values reveals a more drastic discrepancy. Figure 2(b) depicts the difference in the CGI values determined using ICP-OES and XRF as function of the GGI. The error bars stem mostly from the ICP-OES background contamination level, and marginally from the repeatability of XRF measurements. A linear correction to the CGI was extracted, evidenced by the gray line. In the following, the sample composition calibration will differ from that of other Empa publications as follows:

(1) $$\begin{array}{cc} \text{GGI} & =1.03\;\;\cdot \;{\text{GGI}}_{\text{XRF}}\\ \text{CGI} & ={\text{CGI}}_{\text{XRF}}+0.129-0.286\;\cdot \;GGI \end{array}$$

The experimental uncertainty on the raw XRF composition amounts to ±1% on the GGI for GGI values close to 0.35, and ±1.5% on the CGI, plus a systematic error related to the ICP-OES composition correction. A contamination of the CuGaSe_2_ sample with In (GGI 0.99) was evidenced by both ICP-OES and EDX.

### 4.2. Reflectance and transmittance measurements

After transfer onto transparent substrates the CIGS layers were characterized by reflectance and transmittance spectroscopy. An accurate processing of ellipsometry data was impractical because of the CIGS surface roughness visible in the SEM micrograph in Figure 3. For this reason, we report the light absorption coefficient *k* or *α*, and do not report on the real part *n*.

**Figure 4.**
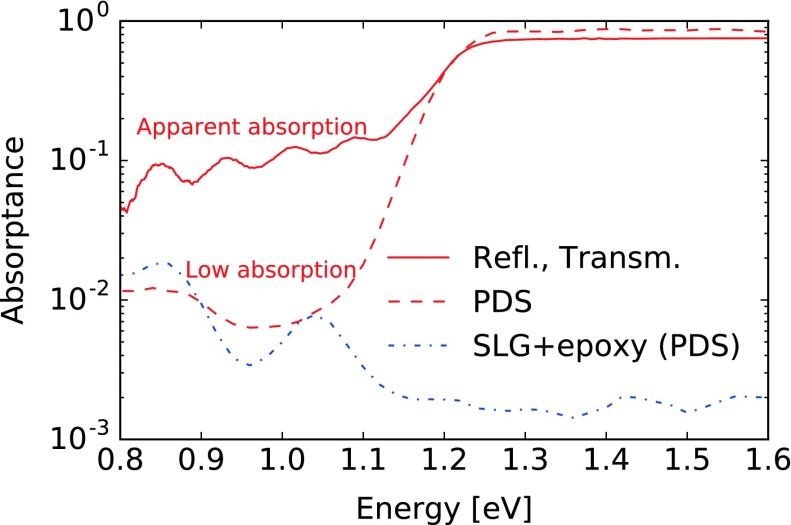
Absorptance spectra of a SLG/epoxy/CIGS multilayer characterized using reflectance and transmittance (continuous lines) and PDS (dashed).

Notes: The discrepancy above 1.25 eV originates in the change in reflectance when the sample is immersed in the liquid. For comparison, the absorptance of an SLG/epoxy sample is shown (dot-dashed).

Reflectance and transmittance spectra indicate an apparent absorptance in the infrared of up to 10%, as shown in Figure 4. This could be interpreted as a residual sub-bandgap absorption in the CIGS, epoxy, or SLG layers. However, in the following we show that the largest contribution originates from light scattering, internal reflection, and light trapping in the multilayer sample. After multiple internal reflections at the interfaces within the sample, the scattered light gets eventually absorbed or escapes the sample sideways, thus avoiding detection in both reflectance and transmittance configurations.

The possibility of light absorption in SLG is ruled out by reflectance and transmittance measurements. Similarly, a smooth epoxy layer on SLG exhibited absorptance below 1% for photon energies above 0.95 eV. By ellipsometry, the refractive index *n* of the epoxy was determined close to 1.57 in the visible range.

Absorbers transferred on SLG or on fused silica substrates were characterized by PDS to investigate the absorption below the CIGS bandgap, as shown in Figure 4 for a typical SLG/epoxy/CIGS multilayer. The sub-bandgap absorptance in the CIGS layer appears around or below 1% far below the bandgap, with detection limited by the parasitic absorption in the SLG/epoxy substrate. As a consequence the apparent absorption observed in reflectance and transmittance measurements is an experimental artifact.

Two experiments were set to verify the light trapping hypothesis and test a procedure which could mitigate this artifact. First, 1 mm-thick single-side and double-side polished fused silica plates were characterized using reflectance and transmittance. The absorptance of the double-side polished plate was below the instrumental detection limit. The single-side polished plates however exhibited an apparent absorptance ranging from 1 to 3% in the IR up to 6% at 400 nm, demonstrating the existence of an experimental artifact caused by light scattering and trapping in the sample.

**Figure 5.**
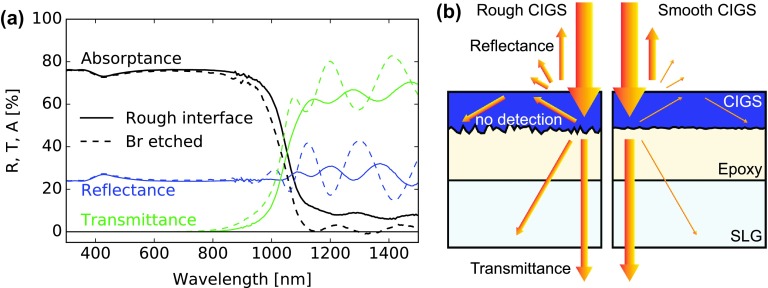
(a) Reflectance, transmittance, and absorptance spectra of a SLG/epoxy/CIGS multilayer without (continuous lines) and with (dashed lines) chemical wet Br polishing (GGI 0.29, CGI 0.82). The horizontal shift in the absorptance curve is caused by the reduced thickness: the absorption curve *α* is essentially unchanged. (b) Schematics of the apparent light absorption caused by the light scattering, internal reflections, and trapping in the layer, for rough and smooth CIGS surfaces.

In a second experiment, a CIGS layer was chemically polished prior to transfer onto transparent substrate following a sequence of wet etchings steps using 10% KCN, an aqueous Br solution as described in Refs. [42,43] and 10% KCN. The initial KCN step improves the homogeneity of the subsequent Br etching, and the final KCN step was applied to remove possible surface residues. A reference piece of the same absorber was treated using a single KCN cleaning step. No change in the layer composition upon preparation could be noticed by XRF. A marked decrease in the surface roughness was observed by visual inspection. Figure 5(a) shows the optical properties of the layers after transfer. The amplitude of the interference fringes markedly increases after etching, and the apparent sub-bandgap absorptance essentially vanishes except for the interferences. This experiment conclusively demonstrates that light can get trapped in the sample during optical measurements and avoids detection in both reflectance and transmission configurations, and that the apparent absorptance observed as large as 10% actually are measurement artifacts. As a general rule, we recommend great care when evaluating absorptance of rough layers from reflectance and transmittance measurements.

For one specific sample, an actual absorption could be observed well below the CIGS bandgap. That sample was grown slightly over Cu stoichiometry (less than 2% relative) and its surface CuSe_x_ compounds were removed by KCN wet etching prior to layer transfer. In spite of this treatment *α* values were unusually large in the infrared (larger than 300 cm^−1^ using PDS, and than 1000 cm^−1^ using reflectance and transmittance).

### 4.3. Analysis of absorption data

Absorption *α* curves were computed from reflectance and transmittance data following the approach of Ritter and Weiser [44], which accounts for light reflection at the layer –substrate interface. The back-reflection parameter *R*_2_ was fixed to 0.1, estimated from the SLG refractive index determined in-house and from that of CIGS from Ref. [24]. The reliability of the *α* values is considered good for values between 1000 and 40,000 cm^−1^, with limiting factors on the one hand the interference fringes and apparent residual absorption, and on the other hand the near complete light absorption.

The fit model to the absorption data *α* consisted of a direct parabolic band transition and an exponential decay at low energy with a similar form as an Urbach tail. The connection energy *E*_*c*1_ and the exponential prefactor *B* are determined by imposing continuity of *α* and of its derivative. The free fit parameters are the optical bandgap *E*_*g*_, a prefactor *A* and the exponential decay energy *U*:

(2) $$\begin{array}{cc} \alpha & =\left\{\begin{array}{cc} B\;\text{exp}\left(\frac{E-E_{g}}{U}\right) & \text{if}\;E<E_{c1}\\ \frac{A}{E}\sqrt{E-E_{g}} & \text{if}\;E\geq E_{c1} \end{array}\right.,\;\text{with}\\ E_{c1} & =\frac{1}{4}\left(2\;E_{g}-U+\sqrt{4\;E_{g}^{2}+12\;E_{g}U+U^{2}}\right)\;\text{and}\\ B & =A\frac{\sqrt{E_{c1}-E_{g}}}{E_{c1}\;\text{exp}\left(\frac{E_{c1}-E_{g}}{U}\right)}. \end{array}$$

The parameters values are analyzed in function of the GGI and CGI values in order to establish an analytical expression for the optical absorption. The bandgap *E*_*g*_ of pure CuInSe_2_ is observed at 1.004 eV and that of pure CuGaSe_2_ is estimated at 1.663 eV by extrapolation. The confidence interval for the reported values is ±0.005 eV, dominated by choices in the fitting procedure. The bowing energy is determined as the best second degree polynomial fit while imposing *E*_*g*_ of pure CuInSe_2_ and CuGaSe_2_ compositions. We propose the following expression for the composition-dependent optical bandgap *E*_*g*_:

(3) $$E_{g}=1.004\;\left(1-\text{GGI}\right)+1.663\;\text{GGI}-0.033\;\text{GGI}\left(1-\text{GGI}\right)$$

For each of the samples the residual error on the bandgap *E*_*g*_ with respect to Equation (3) is within with the composition instrumental uncertainty. We observe no dependency of the residual error on the copper content, i.e. the bandgap appears independent from the Cu content in the investigated CGI range. This is notably in contrast with Ref. [23] which reports a significant increase in the bandgap even for moderate levels of Cu deficiency. We also report a 0.03 ± 0.08 value to the bowing coefficient, lower than the 0.15–0.20 eV values often reported [17,22,45], although the spread in the literature values is wide [45]. This value is very sensitive to systematic errors in the GGI determination. The reported confidence interval stems from a possible nonlinearity in the GGI by ±0.03 at a GGI of 0.50 (somewhat larger than the combined errors of XRF and ICPMS measurements). A meV precision is nevertheless reported in view of later simulations, based on measurements affected by the same systematic error.

**Figure 6.**
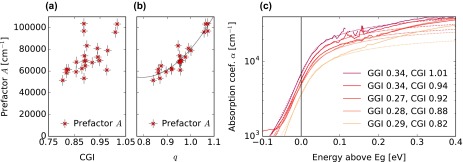
(a) Amplitude prefactor *A* as function of the samples CGI. (b) *A* as function of the coordinate *q*, with *θ* = 0.2076. The line indicates the best parabolic fit. (c) Logarithmic plot of the absorption spectra *α* of samples with comparable GGI and various CGI compositions (continuous lines). For clarity, the curves have been horizontally shifted by setting the fitted optical bandgap *E*_*g*_ to 0. The best fits are shown as dotted lines.

The amplitude prefactor *A* primarily depends on the CGI, however a weaker dependency on GGI is also observed. A new composition plane (0,p,q) is therefore defined by a rotation of the plane (0,GGI,CGI) by an angle *θ*. Thus, the compositional dependency is expressed as a second degree polynomial of a single parameter *q*, with the rotation angle *θ* a fit parameter. The best fit is drawn in Figure 6(b) and was calculated as:

(4) $$\begin{array}{cc} A & =80311\;q+427633\;\left(1-q\right)-596825\;q\left(1-q\right),\;\text{with}\\ q & =\text{GGI}\;\sin\theta +\text{CGI}\;\cos\theta \;\text{and}\\ \theta & =0.2076\;\;\text{rad} \end{array}$$

The equation is valid for CGI values above 0.75. Figure 6(c) illustrates the effect of a change in the prefactor for samples with similar GGI but different CGI compositions. The *α* values are approximately doubled from the sample with the lowest to the sample with the highest CGI value. At higher energies, a comparison of the data to the model based on the parabolic band approximation (dotted lines) reveals the onset of a steeper increase in *α*, initiating around 0.15 eV above *E*_*g*_. This increase is in agreement with earlier experimental [24,46] and simulations results [47].

The observation of a stronger optical absorption with increased CGI is interesting, as it is in line with some observations [14,23] but was often overlooked in previous reports of the CIGS dielectric function [3,20,26]. This hints at an increased density of states in the valence band in the vicinity of the valence band maximum. This interpretation is supported by theoretical studies where the structure of the valence band was shown to be primarily determined by hybridization of Cu d and Se p orbitals [46,48,49]. It must be noted that an increase in the absorption coefficient value can be mistaken for a decrease in the bandgap, especially if few data points are available in its immediate vicinity. A direct consequence of this increase in *A* is a more abrupt long wavelength edge of the EQE, when modifying the compositions of absorbers from Cu-poor toward stoichiometric compositions.

**Figure 7.**
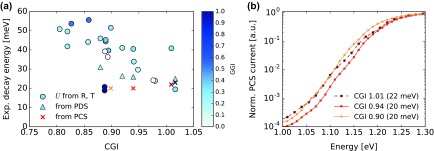
(a) Exponential decay energy as function of the sample CGI, determined from reflectance and transmittance (circles), PDS (triangles), and PCS (crosses) techniques. A trend for higher values is observed for low Cu contents. For most samples, the value of *U* determined from reflectance and transmittance is significantly larger than the Urbach energy decays (see text). (b) Normalized PCS spectra of cells processed from single-stage absorbers with similar GGI and different CGI.

The exponential decay energy *U* is determined from the steepness of the absorption curve *α* in a logarithmic plot, visible for example in Figure 6(c). Values of *U* between 20 and 55 meV are observed and are reported in Figure 7(a). No trend with growth temperature was identified. The large spread in the values is partly caused by the interference fringes in the *α* spectrum at low energies, which can affect the slope of the exponential decay depending on the bandgap and on the sample thickness. Nevertheless, a trend is visible toward decreased decay energies for higher CGI values, hinting to a lesser degree of disorder for compositions close to Cu stoichiometry.

The *U* quantity unfortunately cannot be identified to the Urbach absorption tail energy, because *U* is here characterized barely below the bandgap whereas the Urbach energy should be determined at lower energies. The Urbach tail can be more adequately characterized by PDS, performed in this study on four samples with comparable GGI and different CGI values after CIGS layer transfer onto SLG or fused silica substrates. The exponential decay in the absorptance appears significantly steeper when using PDS than when using reflectance and transmittance, as visible in Figure 4. Depicted in Figure 7(a) as triangles, the values of *U* determined by PDS are somewhat larger than the Urbach energies reported in the literature, which are generally in a range of 18–25 meV [50–52]. The PDS results do not preclude a slight trend for larger values of *U* at low CGI compositions. This would be in qualitative agreement with the trend reported by Shioda et al*.* [53] who attributed the larger Urbach energies observed for low CGI to an increased compositional disorder.

Another estimate to the Urbach energy can be obtained from PCS measurements. Figure 7(b) shows the spectra of three single-stage absorbers with GGI around 0.33 processed into cells, and the corresponding energies are reported in Figure 7(a) together with the sample with Cu excess. These layers are composed of a large number of small grains (see Figure 3). By comparison, PCS measurements on high-efficiency three-stage absorbers with large grains deposited with the same highest temperature resulted in Urbach energy values below 20 meV (not shown). Since PCS is not affected by absorption in the substrate, it provides with a better sensitivity to low absorption levels than PDS on our transferred layers. However, the detection is limited to absorption processes resulting in the collection of the photogenerated charge carriers. Therefore, PCS may provide an estimate to the Urbach energy but is not our technique of choice to determine the absorption tail.

The treatment of reflectance and transmittance data could be refined to some extent. First, by fitting only the exponential decay region the *U* values would be lower by 10–15%. Second, it can be observed in Figure 5(a) that the average level of the transmittance is more affected upon CIGS surface chemical polishing than that of the reflectance. We can consider multiplying the transmittance curve with a constant, such that the computed absorptance would become zero a few hundred meV below the bandgap: such data processing would further decrease the *U* values by around 5–10%. With both corrections *U* would decrease to values compatible with the PDS measurements. A trend for somewhat larger *U* values at low CGI compositions would also remain. Applying these corrections would not significantly affect the values of the optical bandgap *E*_*g*_ and of the prefactor *A*.

Therefore we model the optical absorption *α* below the bandgap as an exponential tail, with decay energy *U* = 25 meV independent from the sample composition due to the lack of more conclusive data.

### 4.4. Expression for the absorption at higher energies

In the previous sections, an expression for the absorption spectrum *α* was established notably assuming a single parabolic band. The investigated layers thicknesses provide the best accuracy close to the bandgap, such that the expression is well suited to model the EQE shape with absorber thicknesses above 1 μm. However, we need a mathematically continuous model also valid at higher energies, especially when modeling thin absorber layers or depth-dependent carrier collection. As can be observed in Figure 6(c), the experimental absorption curves increase faster than the model starting around 0.15 eV above the bandgap. This increase is difficult to adequately describe from our data due to the low transmittance intensity. In the following, we propose an extension of the model presented before based on the work of Minoura et al*.* [24], in which thin absorbers (around 50 nm) deposited on Si substrates were characterized using ellipsometry. Close to the bandgap energy, ellipsometry of such thin layers may result in inaccuracies due to the low level of light absorption, possible composition deviations, the large density of grain boundaries at the lower interface, and possible interactions with the substrate. Nevertheless at higher energies, we expect quite reliable results. Up to 2.5 eV the absorption spectrum *α* described in Ref. [24] follows a rather regular trend. We decide for a polynomial expression with a 1/*E* prefactor by similarity to the analytical form of the single-band approximation. By imposing continuity of both *α* and of its derivative, the connection energy *E*_*c*2_ and an energy shift ∆ are determined, and the following expressions are obtained:

(5) $$\begin{array}{cc} \alpha & =\left\{\begin{array}{cc} \text{Eq}\text{. 2} & \text{if}\;E\leq E_{c2}\\ \frac{C}{E}{\left(E-E_{c2}+\Delta \right)}^{m} & \text{if}\;E>E_{c2} \end{array}\right.,\;\text{with}\\ E_{c2} & =E_{g}+{\left(2^{m}m^{m}\frac{C}{A}\right)}^{-\frac{2}{2\text{m}-1}}\;\text{and}\\ \Delta & ={\left(\frac{A}{C}\sqrt{E_{c2}-E_{g}}\right)}^{\frac{1}{m}}. \end{array}$$

Up to 2.5 eV a reasonable match with the data of Minoura et al. [24] can be obtained with the following parameters set:

(6) $$\begin{array}{c} m=5,\\ C=1.8\;\cdot \;10^{3}\;CGI\left(0.5+0.5\;GGI\right). \end{array}$$

**Figure 8.**
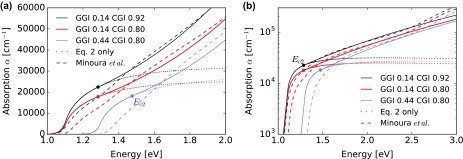
Modeled optical absorption spectra *α* for different CIGS compositions, in (a) linear and (b) logarithmic axis.

Notes: The full expression for *α* is shown with continuous lines, while dotted lines represent *α* from Equation (2) only. The connection energy *E*_*c*2_ is marked with circles. The results from Minoura et al*.* [24] are shown with dashed lines. Close to the bandgap energy, we report significantly larger absorption coefficients than Ref. [24].

Figure 8 shows the absorption spectra *α* for different device-relevant compositions. With this the validity domain of *α* is extended up to 2.5 eV, i.e. down to around 500 nm wavelength. As compared to Minoura’s results, we report significantly larger absorption coefficients in the vicinity of the bandgap, especially for relatively high GGI compositions as shown in Figure 8.

Above 2.5 eV, the absorption spectrum *α* further increases. Together with the counterpart in the real part of the refractive index *n* this results in a feature in the reflectance spectrum visible in Figure 5(a) close to 420 nm.

## 5. Numerical simulations

**Figure 9.**
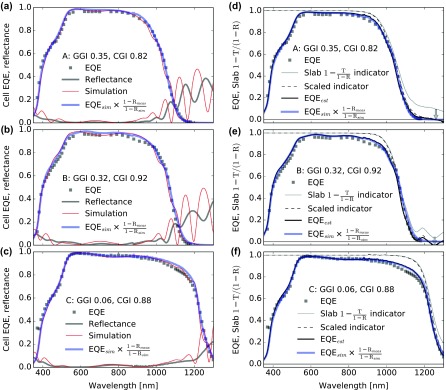
(a), (b), (c) Experimental and simulated EQEs and reflection for samples A, B, and C. Experimental data are shown with gray squares and thick lines and the corresponding simulations with red lines. Simulated EQE corrected with experimental reflectance data are shown with thick blue lines. (d), (e), (f) Experimental EQE (gr ay squares) and estimators (black lines) for samples A, B, and C. Gray lines show the $1-T/\left(1-R\right)$ indicators of absorbers transferred onto transparent substrates, which are then scaled (arrows) and shown with dashed lines. Thick black lines display the final EQE estimator accounting for the experimental cell reflectance and light absorption in the window layers. For a typical CIGS multilayer structure, this estimator appears as reliable to predict the EQE as the simulations, reported from (a) to (c).

In order to validate the dielectric functions determined in this work, three multistage CIGS absorbers with different compositions and optical bandgaps were processed into solar cells and characterized by XRF, ToF-SIMS, EQE, and reflectance. Samples A and B are high-efficiency devices with different CGI compositions, with details available in Ref. [14] (designated as ‘reference’ and ‘17% relative CGI increase’). Sample C is based on a low-bandgap CIGS absorber with Ga grading toward the back interface to improve carrier collection. More details were presented in [54] (designated as ‘BG2’). The experimental active area EQE and reflectance are shown in Figure 9(a)–(c) as gr ay symbols and lines.

**Figure 10.**
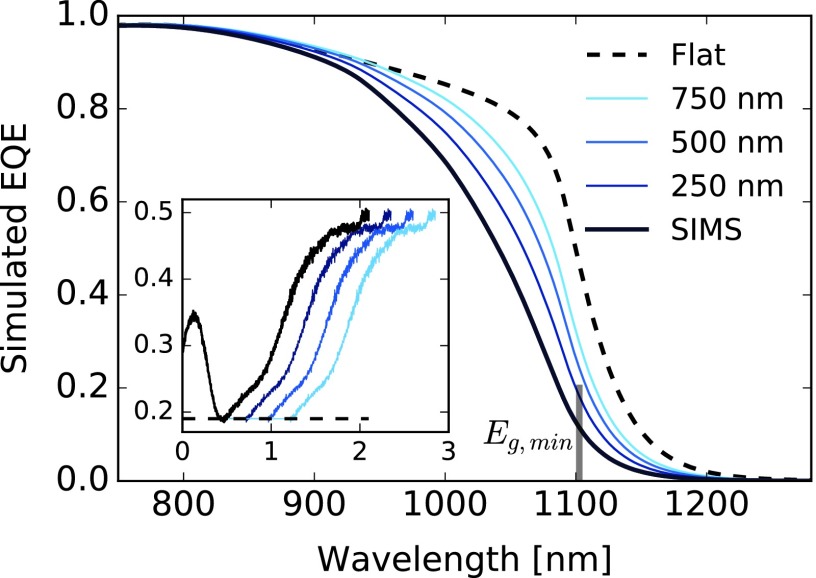
Simulated EQEs based on the SIMS GGI profile of sample A, not corrected for experimental reflectance.

Notes: A flat segment with variable length was added in the notch region to probe the resulting gain in current. A flat GGI profile was also simulated (dashed lines). (inset) GGI profiles vs. absorber depth used as inputs to the simulations.

The optical propagation in the solar cells was simulated using the transfer matrix method (TMM) implemented in the *tmm* python package [55]. The multilayer structure is summarized in Table 3 and can be described as follows: 500 nm Mo, 10 nm roughness layer, 10 nm MoSe_x_, 10 nm roughness layer, CIGS, 35–45 nm CdS, around 65 nm ZnO, around 210 nm ZnO:Al, and 105 nm MgF_2_. Roughness layers were implemented as Bruggeman EMA in a 50–50% mixture of the surrounding materials. For each material, the dielectric functions determined above were used, with the exception of MoSe_x_ for which the values were taken from Evans and Hazelwood [10]. This data-set allows a better agreement with cells reflectance data for energies below the CIGS bandgap. The MoSe_x_ was the only investigated material for which the preparation method differed significantly from that in an actual device.

**Table 3.** Summary of the sample properties, dielectric function data-set and layer thicknesses used for the optical simulations of devices, as presented in Figure 9.

Layer\sample	Data-set	A	B	C
GGI		0.35	0.32	0.06
GGI		0.82	0.92	0.86
Jsc [mA cm^−2^]		35.9	36.5	42.5
MgF_2_ [nm]	This work	105	105	105
ZnO:Al [nm]	This work	200	225	230
ZnO [nm]	This work	60	65	70
CdS [nm]	This work	35	30	45
CIGS [nm]	This work	2.10	2.14	2.70
Roughness [nm]	EMA	10	10	10
MoSe_x_ [nm]	Ref. [10]	10	10	10
Roughness [nm]	EMA	10	10	10
Mo [nm]	This work	500	500	500

Notes: Roughness layers were computed as Bruggeman EMA models [31] in a 50–50% mixture of the surrounding materials. Short-circuit currents are given with ARC and are deduced from active area EQE.

The thicknesses of the window layers are based on typical process values and finely tuned by a few percent to match the fringes observed in the visible range of the reflectance spectra. The CIGS absorbers were modeled as a multilayer of 25 nm thick slices. For each slice, the GGI composition was determined from the ToF-SIMS GGI depth profiles. The CGI composition was assumed uniform and fixed to the integral XRF value. Light absorption in the CIGS layer was computed from the expression of *α* determined above. The real part of the refractive index *n* was taken from the work of Minoura et al*.* [24]. The simulated EQE was obtained by integration of the absorption over all CIGS slices, assuming complete collection of the photogenerated charge carriers. The sample compositions and layer thicknesses are summarized in Table 3. The simulated EQE and reflectance are shown in Figure 9(a)–(c) as thin red lines. At this point, the agreement with the experimental data is reasonable and acceptable for many applications.

At long wavelengths the fringes are shifted as compared to the measurements. Such shifts could be accounted for by slight adjustments of the CIGS thickness (4% or less) that can be justified by some error in the thickness or by process inhomogeneities. These discrepancies might also be caused by the *n* values for CIGS that were taken from Ref. [24]. The reflectance also appears not perfectly predicted in the visible range: the overall shape is qualitatively reproduced but the values are somewhat overestimated. This is likely due to the roughness of the window layers increasing the light penetration in the layers. While in principle this issue can be treated in the framework of the scalar scattering theory [56], a simpler approach was used here. Due to the weak absorption in the window layers, the error on the amount of light entering the absorber corresponds to the error on the reflectance. Thus, a correction factor $\left(1-R_{\text{meas}}\right)/\left(1-R_{\text{sim}}\right)$ is applied to the simulations: the resulting EQE curves (thick blue lines) better match the experimental data. The amplitude of the fringes is also reduced especially at long wavelengths.

The simulations slightly overestimate the experimental EQEs, independently of the wavelength. This observation suggests a current loss mechanism at the absorber –buffer interface or in the window layers. However, the discrepancy is small, such that the experimental errors prevent a quantitative current loss analysis. Only for sample C a marked difference can be distinguished in the EQE curves above 1000 nm. The same analysis was performed for a CuInSe_2_ absorber sample grown with no Ga grading electron reflector at the back contact (not shown here). Compared to sample C, that sample exhibits much larger experimental EQE losses at long wavelengths [54], which illustrates the effectiveness of the Ga grading electron reflector, and allows to ascribe the discrepancy above 1000 nm to an incomplete collection of charge carriers generated deep in the absorber.

We next show that the EQE can also be predicted from reflection and transmission measurements of absorbers if the absorption coefficients of the window layers are known. CIGS absorbers A, B, and C were characterized by transmission and reflection after transfer onto transparent substrates. We estimate the absorption in the CIGS by neglecting the back interface reflection (the largest difference in refractive indices occurs at air –CIGS interface, and the reflection at the CIGS –epoxy interface is hampered by the interface roughness). With *I*_0_ the incident light intensity, an intensity $I_{\text{CIGS}}=I_{0}\left(1-R\right)$ penetrates in the CIGS, and an intensity *I*_0_ *T* is transmitted. It follows that:

(7) $$\text{indicator}=\frac{I_{\text{absorbed,CIGS}}}{I_{\text{CIGS}}}=\frac{I_{0}\;\left(1-R\right)-I_{0}T}{I_{0}\;\left(1-R\right)}=1-\frac{T}{1-R}$$

This indicator of the light absorption ranges from 0 to 1 and is displayed in Figure 9(d)–(f) as a thin gray line. As observed in the case of Br-etched CIGS layers, the transmittance is more affected than the reflectance by scattering and incomplete detection due to rough interfaces. Therefore, the transmission can be scaled linearly such that the value of the indicator becomes zero on average well below the optical bandgap, as shown with a dashed line (for Figure 9 we average in a 300 nm range starting 100 nm above the 0.5 value). This is mathematically equivalent to scaling the indicator over the 0–1 value range, with *avg* the averaged value of the indicator below the bandgap. The scaled indicator can be thought as an estimator to the cell internal quantum efficiency IQE:

(8) $${\text{indicator}}_{\text{scaled}}=\frac{\text{indicator}-\text{avg}}{1-\text{avg}}$$

In the development above the back interface reflections were neglected. The absorption in the CIGS is overestimated when neglecting the reflection at the CIGS –epoxy interface (around 10% from the contrast of refractive indices), but also underestimated when neglecting the reflection at the CIGS–MoSe_x_–Mo interface (10–25% in the vicinity of the bandgap). The result is therefore close to the actual value. If the back interface would be more reflective (but still without strong interferences), a suitable approximation could be obtained by computing an effective absorption curve *α* according to the Ritter’s approach [44], then computing the absorption in the CIGS considering partial reflections at the CIGS interfaces.

The cell EQE is estimated by taking into account the experimental cell reflectance *R*_cell_ as well as the absorption in the window layers. In the layer sequence MgF_2_, ZnO:Al, ZnO, CdS, CIGS, the reflections at interfaces are relatively weak and produce limited interferences, as *n* only increases in the sequence over most of the wavelength range. Therefore, reflections at successive interfaces quickly escape the multilayer and are accounted for in the experimental reflectance. The absorption in the layers can be computed using the Beer–Lambert law according to the layer thicknesses *d*_*i*_. We finally get an estimator for the EQE:

(9) $${\text{EQE}}_{\text{est}}=\frac{\text{indicator}-\text{avg}}{1-\text{avg}}\times \left(1-R_{\text{cell}}\right)\times \prod_{i\;\in \text{window}}\text{exp}\left(-\alpha_{i}{\text{d}}_{i}\right)$$

The estimated EQEs are shown as thick black lines in Figure 9(d)–(f), and match closely both the active area EQE and the simulated EQE corrected for experimental reflectance. When investigating the collection losses of specific cells, this alternative estimator presents several advantages over the simulation method: only reflectance and transmittance measurements are required thus sparing compositional depth profiling (SIMS or equivalent), the hurdle of the CIGS composition calibration is relaxed, and no optical simulation software is required. However, the simulation method is more advantageous when systematically investigating various parameters or when depth profiling can be conducted on a routine basis, as calculations are performed at low cost.

The simulation model can be used for designing solar cells, evaluating alternative materials or optimizing layer thicknesses. In the following, we estimate the light absorption gain caused by variations in the GGI grading. Considering the multilayer and GGI profile of sample A, we expand the notch region by inserting a flat segment with variable length as depicted in Figure 10 (inset). As a limit case we also model an ungraded absorber with same thickness as sample A. The GGI profiles are discretized in 25 nm slices and the cell current *J*_sc_ is computed by integration of the absorption in the CIGS slices, assuming complete carriers collection. The effect of the compositional profile is graphically best evidenced when assuming incoherent propagation in the CIGS layers, canceling the fringes at the EQE edge. The simulated *J*_sc_ of sample A is 36.0 mA cm^−2^. Upon widening the notch region by 250, 500, and 750 nm a respective increase in *J*_sc_ by 0.8, 1.3, and 1.8 mA cm^−2^ is observed. An upper limit to the gain in current is estimated from the flat GGI profile, in this case 2.5 mA cm^−2^. The gains in current reported here might slightly vary according to the specific absorber grading and thickness. The influence of different Ga gradings on the EQE shape is illustrated for example in Ref. [57].

## 6. Conclusions

We used a combination of ellipsometry, reflectance, and transmittance measurements to determine the dielectric functions of the CIGS and other layers forming the front and back contact of a CIGS solar cell. The confidence in the dielectric functions is improved by combining these methods as compared to ellipsometry alone.

Ungraded CIGS layers with various GGI and CGI compositions were characterized by means of reflectance and transmittance. The significant apparent sub-bandgap light absorption is attributed to light scattering at the interfaces and subsequent trapping in the multilayer.

The absorption spectra *α* of the CIGS layers were fitted close to the bandgap energy, and the model parameters expressed as functions of the GGI and CGI layer compositions. Within the investigated composition range, the optical bandgap is determined by the Ga content and it does not depend on the Cu content. By contrast, the absorption coefficient value largely depends on the CGI and to some minor extent on the GGI, which may be attributed to an increase in the density of states close to the valence band maximum for increased Cu contents. The reflectance and transmittance methods were observed not adequate to characterize the low-energy exponential decay, but PDS and PCS techniques can be used instead. An expression for the composition-dependent absorption spectrum *α* is proposed, with validity range down to around 500 nm.

Reflectance and EQE of solar cells were simulated using experimental GGI depth profiles as inputs. The simulations remarkably well reproduced the measured absorption edge, and a detailed comparison enabled to differentiate carrier collection losses from optical absorption losses. For this purpose, an alternative method was developed where optical measurements are performed on absorber layers transferred on transparent substrates. This approach, simple to implement and insensitive to composition miscalibrations, was shown to adequately reproduce the EQE of high-quality devices.

The simulation tool developed herein enables designing multilayer solar structures. As an application example CIGS cells with increased widths of the grading notch were simulated to quantify possible gains in current and to determine the absorption edge.

## Funding

This project has received funding from the Swiss State Secretariat for Education, Research and Innovation [SBFI contract REF-1131-52107] in the framework of the European Union’s Horizon 2020 Research and Innovation Programme [grant agreement number 641004] (‘Sharc25’); the Swiss Federal Office of Energy [SFOE contract SI/501145-01 ‘CIGS25’]; the Competence Center for Energy and Mobility (CCEM) in the ETH-Domain (‘CONNECT-PV’).

## Supplemental data

The supplemental data for this article can be accessed at https://doi.org/10.1080/14686996.2018.1458579

## Disclosure statement

No potential conflict of interest was reported by the authors.

## Supplementary Material

SI.zip
