# Supplementary material for: Refractive indices of layers and optical simulations of Cu(In,Ga)Se2 solar cells
Source: Sci Technol Adv Mater. 2018 May 15;19(1):396–410. doi: 10.1080/14686996.2018.1458579 (PMC5954485; doi:10.1080/14686996.2018.1458579)
Supplement: SI.zip [file TSTA_A_1458579_SM3263.zip › Carron_RefractiveIndices_3rdAdjustments_SI.docx]

****Refractive indices of layers and optical simulations of Cu(In,Ga)Se_2_ solar cells****

Romain Carron^1^, Enrico Avancini^1^, Thomas Feurer^1^, Benjamin Bissig^1^, Paolo A. Losio^2^, Renato Figi^3^, Claudia Schreiner^3^, Melanie Bürki^3^, Emilie Bourgeois^4,5^, Zdenek Remes^6^, Milos Nesladek^4,5^, Stephan Buecheler^1^, Ayodhya N. Tiwari^1^

1 Laboratory for Thin films and Photovoltaics, Empa, Swiss Federal Laboratories for Materials Science and Technology, Ueberlandstrasse 129, 8600 Dübendorf, Switzerland

2 Institute of Computational Physics, Zurich University of Applied Sciences (ZHAW), Technikumstr. 9, 8401 Winterthur, Switzerland

3 Laboratory for Advanced Analytical Technologies, Empa – Swiss Federal Laboratories for Materials Science and Technology, Ueberlandstrasse 129, 8600 Dübendorf, Switzerland

4 Institute for Materials Research (IMO), Hasselt University, Wetenschapspark 1, B-3590 Diepenbeek, Belgium

5 IMOMEC division, IMEC, Wetenschapspark 1, B-3590 Diepenbeek, Belgium

6 Institute of Physics, Academy of Sciences of the Czech Republic, Cukrovarnická 10/112, 162 00 Prague, Czech Republic

****Supplemental information****

# Experimental details

The CIGS layer transfer process on transparent substrate is depicted in Figure SI.1. The sample is first mechanically strengthened by gluing it on top of a SLG substrate. A SLG substrate is then glued on to the CIGS absorber using a transparent epoxy (3M DP100). The epoxy thickness is controlled using polyimide films as spacers. After a suitable curing time the CIGS layers are mechanically peeled off from the Mo-coated substrate.

[Figure SI.1 near here]

ToF-SIMS measurements were performed as follows. The primary beam ions were Bi+ with 25 keV acceleration, total current of 1 pA and a raster size of 100 × 100 μm2. The sputtering beam was a 2 keV, 400 nA O2+ ion source with an on-sample area of 300 × 300 μm2.

Ellipsometry measurements were performed with a Woollam M2000 V-NIR instrument in a 370 nm - 1670 nm wavelength range at incidence angles of 50°, 60° and 70°. Layers were characterized within hours to days after deposition. The samples were stored in low vacuum at room temperature (< 1 mbar).

For ICP-OES measurements the absorber material was mechanically scratched from the area of ca 2 cm^2^ at the Mo/CIGS interface. The matrix elements were subjected to wet chemical extraction by means of 5 ml HNO_3_ 67% Merck suprapure and 4.5 ml H_2_O_2_ 30% Merck suprapure at room temperature. The solutions were diluted to 25 mL in ultrapure water pre eluated plastic vials, with ultrapure water and directly quantified by ICP-OES using certified element standard materials. The measurement and sample preparation accuracy was tested by analysis of ultrapure water-eluated empty deposition vials and standard reference material.

The EQE spectra were recorded by illuminating the sample with a chopped monochromatic light produced from a white light halogen lamp and a LOT MSH-300 monochromator, under around 0.2 sun bias light. The measured current was calibrated against a certified Si solar cell.

For photocurrent spectroscopy (PCS) measurements the solar cells were illuminated using mechanically chopped monochromatic light. The resulting short-circuit photocurrent was amplified using a low noise current preamplifier, measured using a lock-in amplifier and normalized to the flux of incoming photons.

The optical absorptance of CIGS was measured by photothermal deflection spectroscopy (PDS). The sample (CIGS absorber on transparent substrate) was immersed in a liquid with a large temperature dependency of the refractive index (Fluorinert) and illuminated with a mechanically chopped monochromatic light. The spectra were normalized using the PDS signal measured on a highly absorbing reference sample (carbon nanotubes on a glass substrate). Details about the technique and measurement set-up can be found elsewhere [[1](#_ENREF_1), [2](#_ENREF_2)].

[Figure SI.2 near here]

# Carrier density and mobility in ZnO:Al

A fit to the visible to infrared ellipsometry, reflectance and transmittance measurements of TCO allows estimating the intra-grain mobility and the carrier density [[3](#_ENREF_3), [4](#_ENREF_4)]. Using a dielectric function model composed of a single Lorentz oscillator with $\omega_{0}$ = 0 cm^-1^, best fits to the ZnO:Al data delivered a plasma frequency $\omega_{p}$ values of around 9000 cm^-1^ and a damping frequency $\gamma$ value of around 600 cm^-1^. Assuming $m^{*}$ = 0.28 $m_{0}$ [[3](#_ENREF_3)] and $\varepsilon_{\infty}$= 3.75, one finds a free carrier density $N_{optical}$ = 2.5·10^20^ cm^-3^, in reasonable agreement with the typical $N_{Hall}$ = 3.5 - 4.0·10^20^ cm^-3^ obtained from Hall measurements on comparable layers. The optical mobility $\mu_{optical}$ = 55 cm^2^ V^-1^ s^-1^ was found larger than the Hall mobility $\mu_{Hall}$ = 18 - 20 cm^2^ V^-1^ s^-1^, although similar discrepancies were already reported and explained [[5](#_ENREF_5)].

# Determination of the optical bandgap from the EQE edge

As visible in Figure 10 the EQE absorption edge shifts when the notch width in increased, in spite of modeling an identical bandgap minimum value in each cases. This case study allows for comparing different methods for the determination of the optical bandgap to the actual simulated bandgap minimum. The optical bandgap is often deduced from a linear extrapolation in the $\left( E\cdot EQE \right)^{2}\text{ vs }E$ graph. The grading of sample A leads to an optical bandgap around 18 meV above the minimum of the electronic bandgap $E_{g}$, while for the ungraded cell this is around 15 meV below the minimum $E_{g}$. This last value is possible as the EQE edge is there dominated by absorption in tail states. Another method to determine the cell optical bandgap is the inflection point in the EQE curve (or peak energy of the derivative). This method leads to overestimations of 26 meV for the unmodified profile, and of 3 meV for the ungraded cell. Based on these numbers we conclude the $\left( E\cdot EQE \right)^{2}\text{ vs }E$ method yields lower values of the optical bandgap than the derivative method (here by about 15 meV), and that its results is slightly more dependent on the absorber grading profile. In the case of CIGS however, the derivative method sometimes cannot be applied as interferences fringes heavily distort the shape of the EQE derivative.

[Figure SI.3 near here]


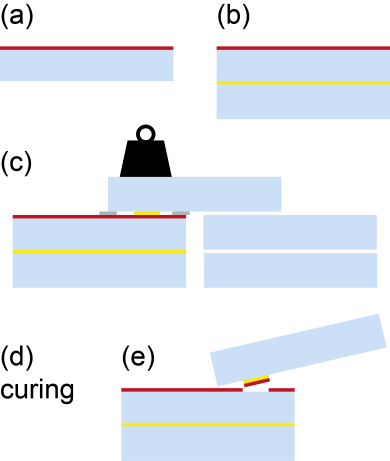


Figure SI.1: Schematic description of the CIGS layer transfer on a transparent substrate. (a) Sample, (b) mechanical strengthening, (c) gluing of a SLG substrate on the CIGS layer using polyimide foils as spacers, (d) curing, and (e) mechanical peel-off.

**
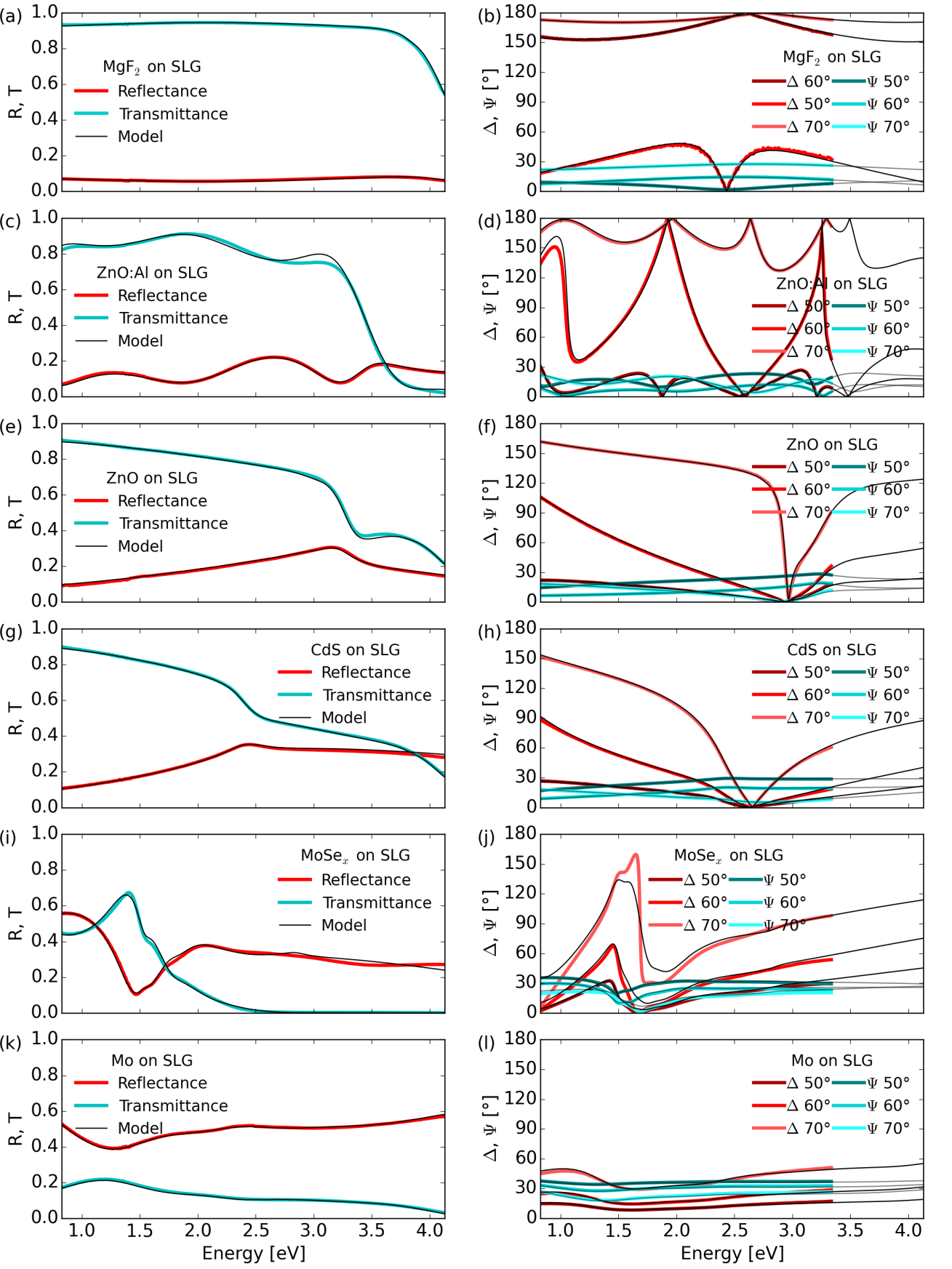
**

**Figure SI.2: Raw data and best fits for different samples deposited on SLG substrates: MgF_2_, ZnO:Al, ZnO, CdS, MoSe**_x_ **and Mo. Reflectance and transmittance are shown on the left hand side panels, and the ellipsometry** $\Delta$ **and** $\Psi$ **spectra acquired at 50°, 60° and 70° incidence angles are shown in the right hand side panels, together with the best fits (see main text).**


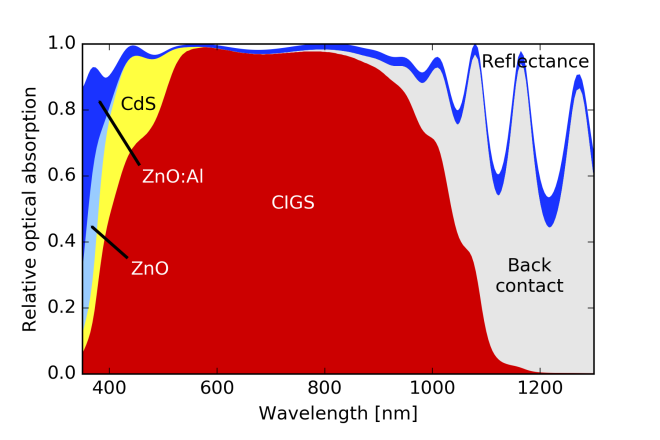


Figure SI.3: Simulated reflectance and optical absorption in the different layers of sample A. The parasitic absorption losses in the window layers amount to: ZnO:Al 1.3 mA/cm^2^, ZnO 0.22 mA/cm^2^, CdS 1.6 mA/cm^2^, and back contact layers 4.7 mA/cm2 (integration range 350-1200 nm). The simulated EQE current is 36.0 mA/cm^2^.

1. Goris L, Haenen K, Nesladek M, Wagner P, Vanderzande D, De Schepper L, D'Haen J, Lutsen L, Manca JV. Absorption phenomena in organic thin films for solar cell applications investigated by photothermal deflection spectroscopy. J Mater Sci. 2005;40:1413-8.

2. Remes Z, Vasudevan R, Jarolimek K, Smets AHM, Zeman M. The optical spectra of a-Si:H and a-SiC:H thin films measured by the absolute photothermal deflection spectroscopy (PDS). Solid State Phenomen. 2014;213:19-+.

3. Steinhauser J, Fay S, Oliveira N, Vallat-Sauvain E, Ballif C. Transition between grain boundary and intragrain scattering transport mechanisms in boron-doped zinc oxide thin films. Appl Phys Lett. 2007;90.

4. Brehme S, Fenske F, Fuhs W, Nebauer E, Poschenrieder M, Selle B, Sieber I. Free-carrier plasma resonance effects and electron transport in reactively sputtered degenerate ZnO : Al films. Thin Solid Films. 1999;342:167-73.

5. Agashe C, Kluth O, Hupkes J, Zastrow U, Rech B, Wuttig M. Efforts to improve carrier mobility in radio frequency sputtered aluminum doped zinc oxide films. J Appl Phys. 2004;95:1911-7.
